# A comparative study of mono-exponential and advanced diffusion-weighted imaging in differentiating stage IA endometrial carcinoma from benign endometrial lesions

**DOI:** 10.1007/s00432-024-05668-8

**Published:** 2024-03-20

**Authors:** Hai-Jiao Li, Kun Cao, Xiao-Ting Li, Hai-Tao Zhu, Bo Zhao, Min Gao, Xiang Song, Ying-Shi Sun

**Affiliations:** 1 Key Laboratory of Carcinogenesis and Translational Research (Ministry of Education/Beijing), Department of Radiology, Peking University Cancer Hospital and Institute, No. 52 Fu Cheng Road, Hai Dian District, Beijing, 100142 China; 2 Key Laboratory of Carcinogenesis and Translational Research (Ministry of Education/Beijing), Department of Gynecological Oncology, Peking University Cancer Hospital and Institute, No. 52 Fu Cheng Road, Hai Dian District, Beijing, 100142 China; 3 Siemens Healthineers Digital Technology (Shanghai) Co., Ltd, Customer Services CRM, No.7 Wangjing Zhonghuan Nanlu, Beijing, 100102 China

**Keywords:** Endometrial neoplasms, Endometrial hyperplasia, Diffusion-weighted magnetic resonance imaging

## Abstract

**Purpose:**

The purpose of the current investigation is to compare the efficacy of different diffusion models and diffusion kurtosis imaging (DKI) in differentiating stage IA endometrial carcinoma (IAEC) from benign endometrial lesions (BELs).

**Methods:**

Patients with IAEC, endometrial hyperplasia (EH), or a thickened endometrium confirmed between May 2016 and August 2022 were retrospectively enrolled. All of the patients underwent a preoperative pelvic magnetic resonance imaging (MRI) examination. The apparent diffusion coefficient (ADC) from the mono-exponential model, pure diffusion coefficient (D), pseudo-diffusion coefficient (D*), perfusion fraction (f) from the bi-exponential model, distributed diffusion coefficient (DDC), water molecular diffusion heterogeneity index from the stretched-exponential model, diffusion coefficient (Dk) and diffusion kurtosis (K) from the DKI model were calculated. Receiver operating characteristic (ROC) analysis was used to evaluate the diagnostic efficiency.

**Results:**

A total of 90 patients with IAEC and 91 patients with BELs were enrolled. The values of ADC, D, DDC and Dk were significantly lower and D* and K were significantly higher in cases of IAEC (*p* < 0.05). Multivariate analysis showed that K was the only predictor. The area under the ROC curve of K was 0.864, significantly higher compared with the ADC (0.601), D (0.811), D* (0.638), DDC (0.743) and Dk (0.675). The sensitivity, specificity and accuracy of K were 78.89%, 85.71% and 80.66%, respectively.

**Conclusion:**

Advanced diffusion-weighted imaging models have good performance for differentiating IAEC from EH and endometrial thickening. Among all of the diffusion parameters, K showed the best performance and was the only independent predictor. Diffusion kurtosis imaging was defined as the most valuable model in the current context.

**Supplementary Information:**

The online version contains supplementary material available at 10.1007/s00432-024-05668-8.

## Introduction

The differential diagnosis of early-stage endometrial carcinoma (EC) from benign endometrial lesions (BELs) is challenging for gynaecologists. Endometrial carcinoma is one of the top three malignancies in women globally, with an increasing incidence (Zhang et al. 2019). The recognition of EC can help with its management, particularly in the early stages. Furthermore, many benign pathologies, such as endometrial hyperplasia (EH) and a physiologically thickened endometrium, may involve the uterine cavity. The clinical manifestations of early-stage EC and BELs have many overlaps, which make a differential diagnosis challenging. In clinical practice, endometrial biopsy and dilation and curettage play an important role in the diagnosis of uterine cavity lesions. However, these procedures have limitations in that they are invasive, can cause complications and discomfort and are nondiagnostic in some cases (Xie et al. 2018). They may also lead to misdiagnoses due to sampling errors (Hanegem et al. 2016). Thus, reliable non-invasive methods are required to assist the preoperative diagnosis.

Magnetic resonance imaging (MRI) is commonly used in the evaluation of endometrial lesions because of its excellent soft tissue contrast resolution, and the method may help in identifying myometrial invasion and cervical involvement. However, there are many reports of overlapping features of benign and malignant lesions (Takeuchi et al. 2005; Manfredi et al. 2005); conventional MRI appears to be of low value in differentiating malignant from benign endometrial lesions, particularly for stage IA endometrial carcinomas (IAECs) and BELs (Natarajan et al. 2020). In IAECs, cancer is present in the endometrium only or less than halfway through the myometrium (the muscle layer of the uterus). To date, there is little consensus on the use of MRI in the routine preoperative assessment of endometrial malignancy.

Diffusion-weighted imaging (DWI), a non-invasive functional MR technique, can be used to diagnose endometrial lesions. There are many diffusion models. The apparent diffusion coefficient (ADC) value derived from the mono-exponential model describes the free diffusion of water molecules in a Gaussian distribution. It is easy to obtain and is the most widely used model in clinical practice. However, it can be influenced by many factors, such as barriers and microperfusion. Therefore, several advanced diffusion models have been used, such as intravoxel incoherent motion (IVIM) and diffusion kurtosis imaging (DKI). Intravoxel incoherent motion includes bi-exponential and stretched-exponential models and is used to reflect capillary microcirculatory perfusion, while DKI describes tissue heterogeneity. The IVIM model was previously found to be related to pathological indicators and useful for the disease grading of EC (Ma et al. 2023; Satta et al. 2021; Chryssou et al. 2022); it also showed higher efficacy than ADC in differentiating an endometrial malignancy from the normal endometrium (Liu et al. 2016). Diffusion kurtosis imaging is based on the non-Gaussian model and has a high sensitivity for reflecting the complexity of the tissue microstructure (Jensen et al. 2005). These diffusion models have been used to evaluate the grading and risk factors of endometrial cancer (Yamada et al. 2019; Li et al. 2021; Chen et al. 2017; Jin et al. 2022); however, these studies were not targeted at early-stage EC. The clinical role of diffusion MRI in the differentiation of IAEC from BELs remains uncertain, and there is no agreement among the existing studies. Therefore, the purpose of this study was to evaluate the use of different MRI diffusion models for differentiating IAEC from BELs.

## Materials and methods

### Population

The Institutional Review Board approved the protocol for this retrospective study and the requirement for informed consent was waived.

**Fig. 1:**
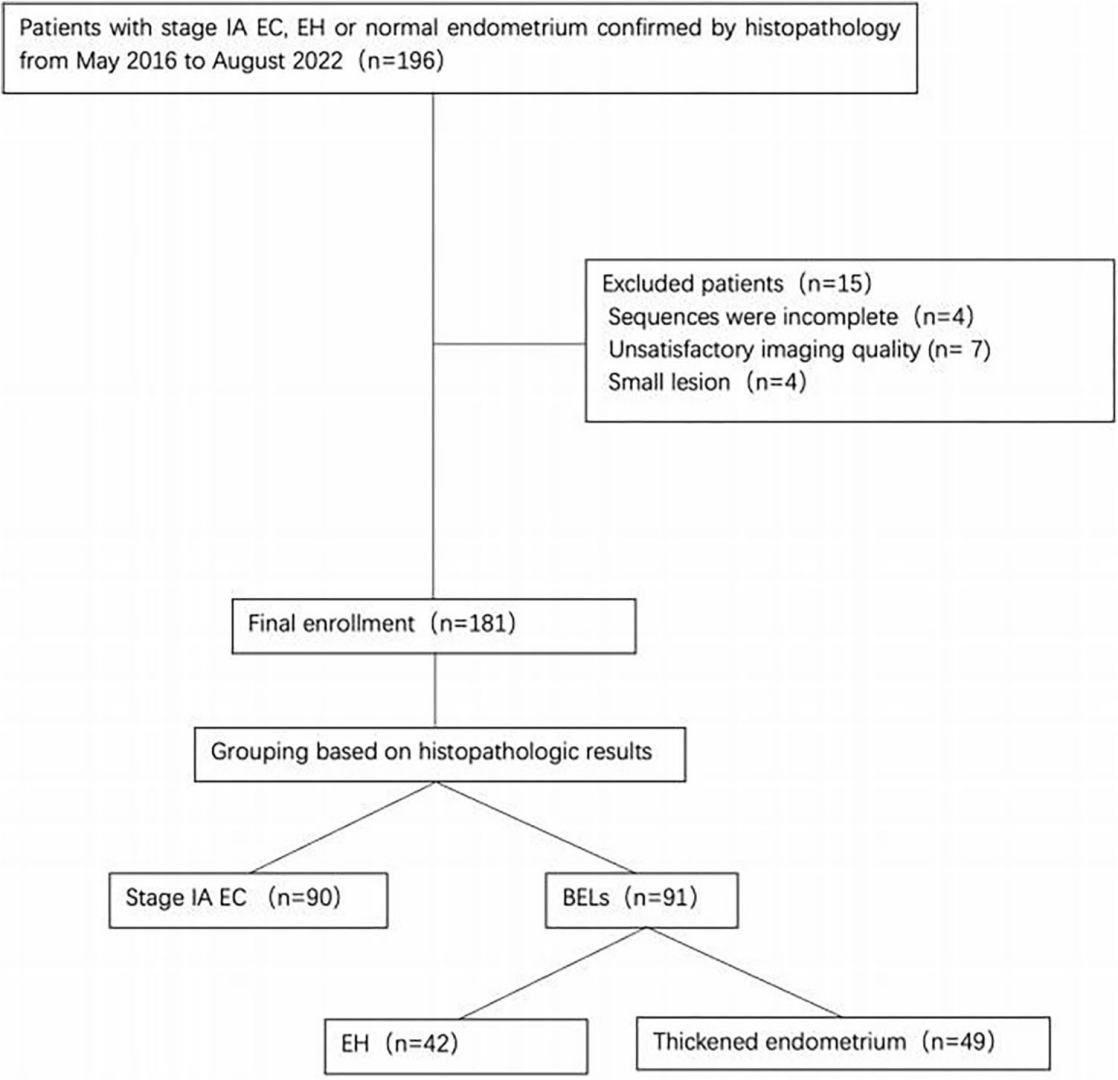
Flow chart of patient enrollment. *EC* endometrial carcinoma, *EH* endometrial hyperplasia, *BELs* benign endometrial lesions

Consecutive female patients with pathologically confirmed IAEC, EH or a physiological thickened endometrium were retrospectively recruited between May 2016 and August 2022 with the following inclusion criteria: (1) a pelvic MRI was performed on the same 1.5T MRI machine, including the same multi-b-value diffusion sequence; (2) the MRI was performed within 20 days before the gynaecological surgery or curettage; (3) all patients had no history of systemic chemotherapy or pelvic radiotherapy. The exclusion criteria were as follows: (1) the quality of the MR image was poor or could not be processed by the software used; (2) the endometrium was too thin (maximum thickness was < 5 mm on sagittal T2-weighted images) for an assessment using MRI (Fig. 1).

### Magnetic resonance imaging protocol

*Conventional MRI scanning* The MRI was performed using a 1.5T MRI scanner (Aera; Siemens Medical Solutions, Erlangen, Germany) with a pelvic phased-array coil. Patients received an intramuscular injection of 10 mg raceanisodamine hydrochloride approximately 20 minutes before the MRI to reduce bowel movement. Patients were in the supine position and allowed to breathe freely during the data acquisition. The following sequences were obtained: axial turbo spin-echo (TSE) T1-weighted imaging (TR/TE = 400–450/8–9 ms, slice thickness/slice gap = 5/1 mm, matrix size = 384 × 384, field of view [FOV] = 26–28 cm), axial TSE T2-weighted imaging (TR/TE = 3000–3500/125–130 ms, slice thickness/slice gap = 5/1 mm, matrix size = 384 × 384, FOV = 26–28 cm), sagittal TSE T2-weighted imaging (TR/TE = 3000–3500/95–100 ms, slice thickness/slice gap = 4/1 mm, matrix size = 384 × 312, FOV = 26–28 cm), axial gadolinium-enhanced fat-saturated T1WI (TR/TE = 6.8/2.4 ms, slice thickness = 3 mm, matrix size = 320 × 195, FOV = 24–28 cm).

*Diffusion-weighted imaging, IVIM and DKI* Axial multi-b-value DWI was performed using a single-shot echo planar imaging sequence during free breathing. The imaging parameters were as follows: TR/TE = 6000 ms/minimum, slice thickness/slice gap = 5/1 mm, matrix size = 256 × 192, FOV = 36–40 cm, number of excitations = 2–3. Eleven b-values were used (b = 0, 20, 50, 100, 150, 200, 500, 800, 1000, 1300 and 1600 s/mm^2^). The total scan time was 6–7 min.

### Imaging processing and analysis

**Fig. 2:**
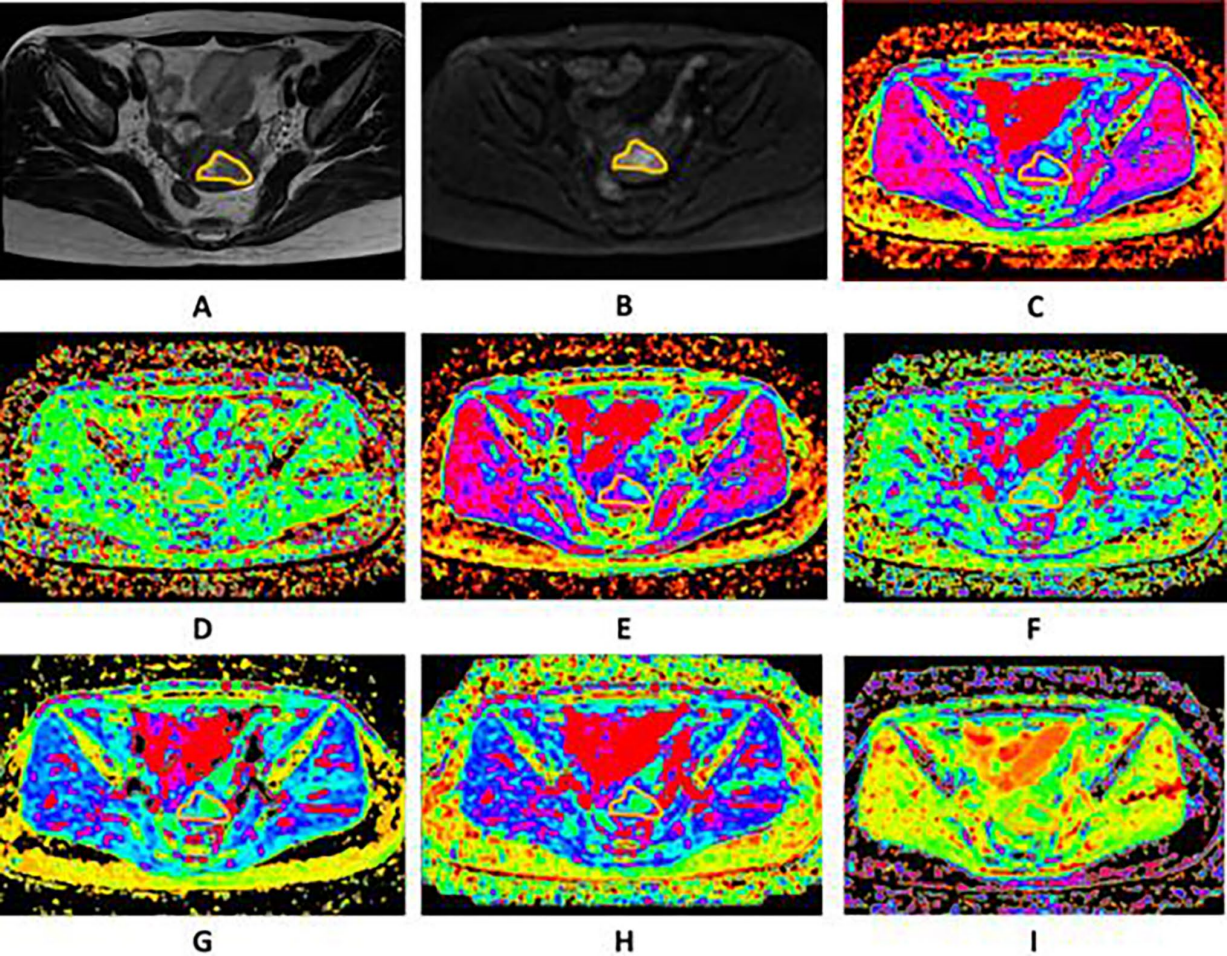
Images of a 57-year-old woman pathologically proven endometrial carcinoma. **A** Axial T2WI shows a mass in the uterine cavity with intermediate signal intensity, with intact myometrium. The ROI was delineated along the outer edge of the tumor on T2WI with reference to diffusion images with b of 1000 (**B**) and was directly co-localized on all parametric maps. **C**–**I** The ADC (**C**), D* (**D**), D (**E**), f (**F**), DDC (**G**), Dk (**H**), K (**I**) were calculated

**Fig. 3:**
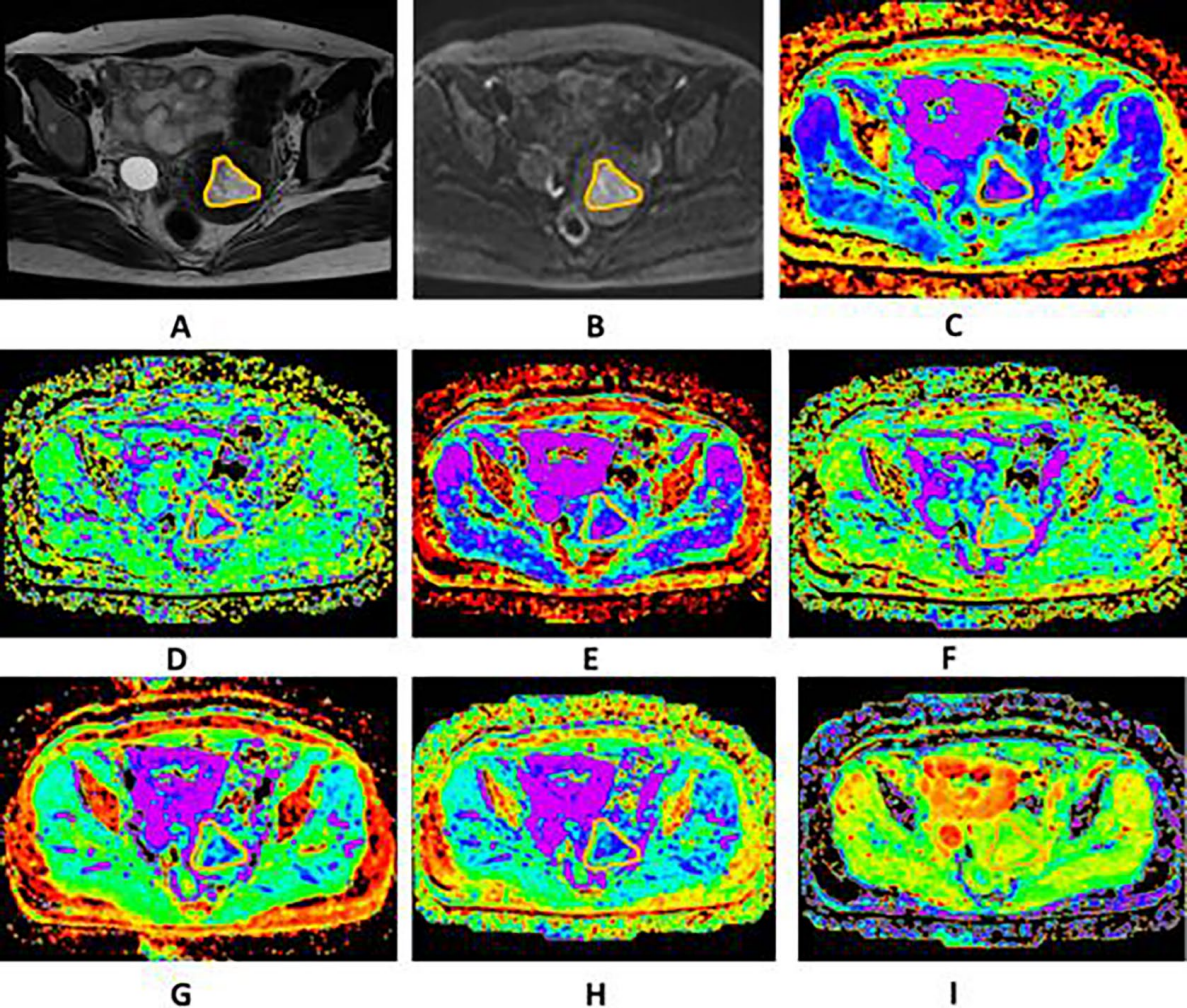
Images of a 39-year-old woman pathologically proven atypical endometrial hyperplasia. **A** Axial T2WI shows a mass in the uterine cavity with intermediate signal intensity, no evidence of myometrium invasion was observed. The ROI was delineated along the outer edge of the tumor on T2WI with reference to diffusion images with b of 1000 (**B**) and was directly co-localized on all parametric maps. **C**–**I** The ADC (**C**), D* (**D**), D (**E**), f (**F**), DDC (**G**), Dk (**H**), K (**I**) were calculated

The parameters calculated from the different DWI models were as follows:

1. The value of ADC was calculated by fitting the signal intensities of 11 b-values (b = 0–1600 s/mm^2^) pixel-by-pixel by linear regression to the mono-exponential model of the DWI using the following equation:
  $${\text{Sb}}/{\text{S}}0 = \exp \left( { - {\text{b}}\cdot{\text{ADC}}} \right),$$
  where Sb and S0 are the signal intensities in the diffusion gradient factors of b and 0, respectively.
2. The bi-exponential model was calculated using the following equation:
  $${\text{Sb}}/{\text{S}}0 = \left( {1 - f} \right)\exp \left( { - {\text{bD}}} \right) + f\exp \left( { - {\text{b}}\left( {{\text{D}} + {\text{D}}*} \right)} \right),$$
  where Sb and S0 are the signal intensities in the diffusion gradient factors of b and 0, respectively; D, the true diffusion coefficient, is the pure molecular diffusion; D*, the pseudo-diffusion coefficient, reflects the incoherent movements of microvascular blood within the voxel; and f, the perfusion fraction, represents the volume fraction of random microcirculation over the total incoherent signal in each voxel.
3. According to the stretched-exponential DWI model, the water molecular diffusion heterogeneity index (α) and the distributed diffusion coefficient (DDC) were calculated using the following formula:
  $${\text{Sb}}/{\text{S}}0 = \exp \left( { - {{\left( {b\,{\text{DDC}}} \right)}^\alpha }} \right),$$
  where DDC represents the mean intravoxel diffusion rate, and α is related to the intravoxel water molecular diffusion heterogeneity, which is bound between 0 and 1.
4. The DKI parameters, including the diffusion coefficient (Dk) and diffusion kurtosis (K), were calculated using the following equation:
  $${\text{Sb}}/{\text{S}}0 = \exp \left( { - {\text{b}}\,{\text{Dk}} + {{\text{b}}^2}{\text{D}}{{\text{k}}^2}{\text{K}}/6} \right),$$
  where Dk and K reflect the diffusion coefficient corrected for non-Gaussian bias and the degree of deviation from the Gaussian distribution, respectively. The four models of DWI processing were performed using custom-written scripts in MatLab (v. R2016a; MathWorks, Natick, MA, USA) to provide ADC, D, D*, f, DDC, α, Dk and K parametric maps on a pixel-by-pixel basis. The lsq_nonlin algorithm, a least squares non-linear fitting method, was employed due to its robustness and accuracy in estimating diffusion parameters from MRI data. This algorithm was implemented with standard parameter settings, as are commonly used in the literature for similar types of analysis. This method was chosen for its efficiency in handling the dataset’s complexity and its ability to provide reliable estimates under the study time constraints. For each patient, two radiologists (LHJ and CK, with 5 and 15 years of experience in gynaecological radiology, respectively), who were blinded to the pathological results, independently drew the volume of interest (VOI) covering the entire tumour along the outer edge of the tumour’s solid components on the DWI at a b-value of 1000 s/mm^2^ with reference to T2WI and contrast-enhanced T1-weighted images using the 3D slicer- software (v.4.8; http://www.slicer.org). Care was taken to avoid the cystic, haemorrhagic and necrotic areas and adjacent normal tissue. The VOIs were copied and pasted onto other parametric maps (including the ADC, D, D*, f, DDC, α, Dk and K maps) using the same software, and the corresponding parameters were obtained. Two examples are shown in Figs. 2 and 3.

### Statistical analysis

Statistical analyses were performed using the SPSS (version 22.0) and MedCalc (version 19.6) software. All parameters were tested using the Shapiro–Wilk test to determine normality. Inter-observer reliability between the two radiologists was assessed using the intra-class correlation coefficient (ICC). Parameters with an ICC > 0.75 indicated good agreement and were selected for further statistical analysis. The independent sample *t*-test and Mann–Whitney U test were used to compare the parameters between groups. Logistic regression analyses were used to identify independent factors. Receiver operating characteristic (ROC) analysis was performed to assess the diagnostic performance for each parameter. The sensitivity, specificity and accuracy at the cut-off values were calculated, and the area under the ROC curve (AUC) was estimated for each parameter. The AUCs of different parameters were compared using the DeLong test. A *p-*value < 0.05 was considered statistically significant.

## Results

### Patient characteristics

A total of 181 patients were enrolled in the study, including 90 patients with IAEC and 91 patients with BELs (42 patients with EH and 49 patients with a thickened endometrium). The mean age of the participants was 48.98 ± 10.32 years, and the median tumour volume was 4.72 (2.99, 8.70) cm^3^. There was no statistically significant difference in the basic data of patients with IAEC and those with BELs, which could be used for subsequent comparisons.

### Reliability of different diffusion parameters

All parameters measured by the two readers showed excellent intra-observer reliability, with ICCs ranging from 0.761 to 0.997 (Supplementary material 1).

### Comparison of different diffusion parameters between stage IA endometrial carcinoma and benign endometrial lesions

The results of the different diffusion parameters of IAEC and BELs are shown in Tables 1 and 2. The ADC, D, DDC and Dk values were significantly lower, and the D* and K values were significantly higher in the IAEC than in the BELs group (all *p* < 0.05). There were no significant differences in the values of f and α between groups (all *p* > 0.05). Univariate analysis showed that D, D*, DDC, Dk and K were independent predictors, while multivariate analysis revealed K as the only independent predictor (*p* < 0.001).

**Table 1:** Comparison of parameters between stage IAEC and benign endometrial lesions

Diffusion model	parameter	Stage IA EC	BELs	t/Z	*p*
Mono-exponential	ADC (× 10^−3^mm^2^/s)	1.06 (0.92, 1.18)	1.09 (0.99, 1.22)	− 2.34	0.001^a^
Bi-exponenti^a^l	D (× 10^−3^mm^2^/s)	0.70 (0.62, 0.78)	0.84 (0.77, 0.94)	− 7.23	< 0.001^a^
	D* (× 10^−3^mm^2^/s)	4.54 (4.25, 4.92)	4.33 (3.70, 4.71)	− 3.20	0.001^a^
	f (%)	2.51 (2.24, 2.87)	2.42 (2.13, 2.76)	− 1.49	0.137^a^
Stretched exponential	DDC (× 10^−3^mm^2^/s)	1.07 (0.91, 1.20)	1.24 (1.15, 1.36)	− 5.64	< 0.001^a^
	α	0.63 (0.59, 0.70)	0.63 (0.57, 0.67)	− 1.02	0.306^a^
DKI	Dk (× 10^−3^mm^2^/s)	1.43 (1.27, 1.60)	1.53 (1.42, 1.65)	− 4.07	< 0.001^a^
	K	0.98 ± 0.15	0.76 ± 0.14	10.26	< 0.001^b^

*EC* endometrial carcinoma, *BEL* benign endometrial lesion, *ADC* apparent diffusion coefficient, *DKI* diffusion kurtosis imaging, *EPI* echo planar imaging, *D* true diffusion coefficient, D^*^ pseudodiffusion coefficient, *f* the perfusion fraction, *α* diffusion heterogeneity index, *DDC* distributed diffusion coefficient, *Dk* diffusion coefficient, *K* diffusion kurtosis, *a* Z, *b* t

**Table 2:** Univariate and multivariate analyses for differentiating stage IAEC and BELs

parameter	Univariate analysis OR (95%CI)	*p*	Multivariate analysis OR (95%CI)	*p*
ADC (× 10^−3^mm^2^/s)	1.524 (0.848–2.739)	0.159		–
D (× 10^−3^mm^2^/s)	8.132 (4.184–15.807)	< 0.001^*^	2.283 (0.531–9.826)	0.267
D* (× 10^−3^mm^2^/s)	0.478 (0.264–0.865)	0.015^*^	0.700 (0.327–1.497)	0.358
f (%)	0.574 (0.318–1.033)	0.064		–
DDC (× 10^−3^mm^2^/s)	3.871 (2.090–7.170)	< 0.001^*^	0.426 (0.080–2.260)	0.316
α	0.820 (0.457–1.469)	0.504		–
Dk (× 10^−3^mm^2^/s)	2.635 (1.446–4.802)	0.002^*^	1.288 (0.395–4.201)	< 0.675
K	0.062 (0.030–0.128)	< 0.001^*^	0.079 (0.026–0.244)	< 0.001^*^

*EC* endometrial carcinoma, *BEL* benign endometrial lesion, *ADC* apparent diffusion coefficient, *D* true diffusion coefficient, D^*^ pseudodiffusion coefficient, *f* the perfusion fraction, *α* diffusion heterogeneity index, *DDC* distributed diffusion coefficient, *Dk* diffusion coefficient, *K* diffusion kurtosis, *p < 0.05

### Diagnostic efficacy of diffusion parameters in differentiating stage IA endometrial carcinoma and benign endometrial lesions

**Fig. 4:**
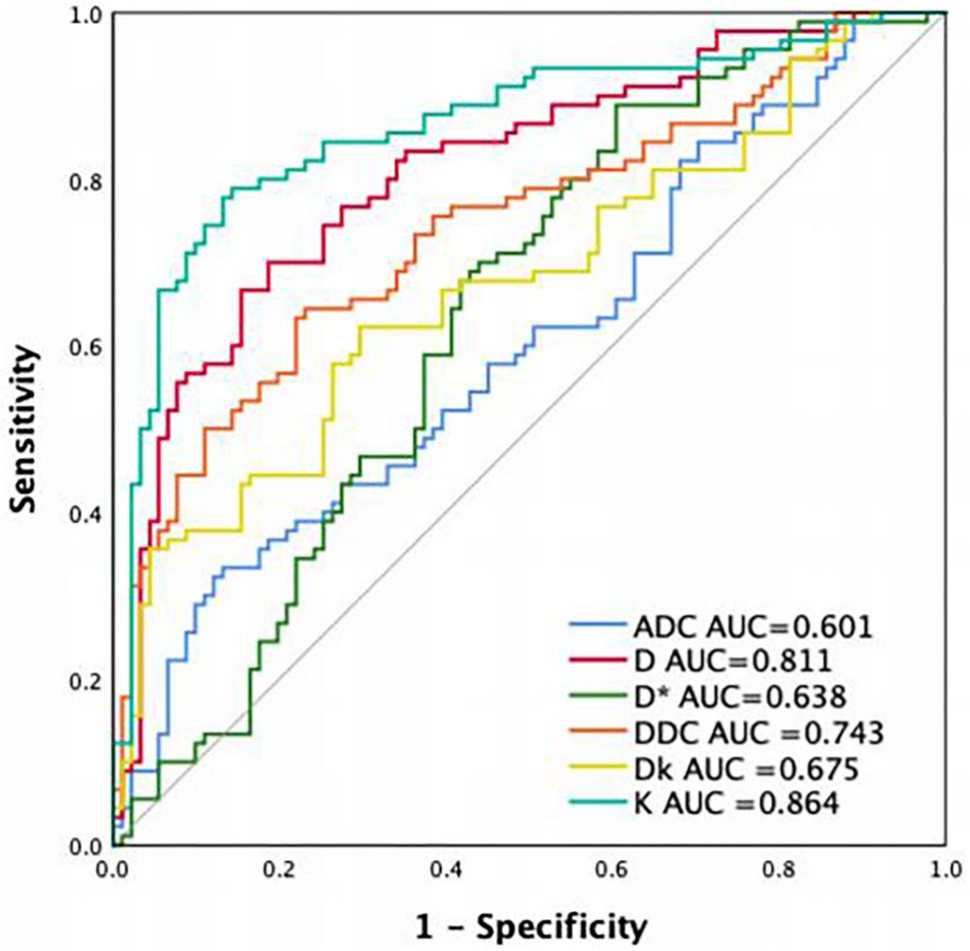
Receiver operating characteristic curves of different diffusion parameters

The results of the ROC curve analysis are summarised in Table 3. For the differentiation of IAEC from BELs, K showed the highest AUC (AUC = 0.864; 95% CI 0.808–0.919; cut-off value = 0.88; sensitivity = 78.89%; specificity = 85.71%; accuracy = 80.66%), followed by D, ADC, DDC, Dk and D* (AUC = 0.811, 0.799, 0.743, 0.675 and 0.638, respectively) (Fig. 4). The AUC of K was statistically higher than that of ADC, D, D*, DDC and Dk (*p* < 0.05). The AUC of ADC was the lowest, and lower than that of D, DDC, Dk and K (*p* < 0.05) (Supplementary material 2). The above results indicated that K exhibited the best performance among all the diffusion parameters.

**Table 3:** The AUC, senseitivity, specificity, and accuracy of parameters

Diffusion model	parameter	Cut-off	AUC (95% CI)	Sensitivity	Specificity	Accuracy
Mono-exponential	ADC (× 10^−3^mm^2^/s)	0.95	0.601 (0.519–0.683)	33.33	86.81	55.25
Bi-exponential	D (× 10^−3^mm^2^/s)	0.76	0.811 (0.748–0.884)	70.00	81.32	74.03
	D* (× 10^−3^mm^2^/s)	4.04	0.638 (0.556–0.719)	88.89	39.56	60.22
Stretched-exponential	DDC (× 10^−3^mm^2^/s)	1.13	0.743 (0.671–0.815)	64.44	76.92	66.30
DKI	Dk (× 10^−3^mm^2^/s)	1.48	0.675 (0.597–0.754)	62.22	70.33	62.43
	K	0.88	0.864 (0.808–0.919)	78.89	85.71	80.66

*ADC* apparent diffusion coefficient, *D* true diffusion coefficient, D^*^ pseudodiffusion coefficient, *f* the perfusion fraction, *α* diffusion heterogeneity index, *DDC* distributed diffusion coefficient, *Dk* diffusion coefficient, *K* diffusion kurtosis, *AUC* area under the ROC curve, *DKI* diffusion kurtosis imaging

### Comparison of different diffusion parameters between subgroups

The values of ADC, D, DDC, Dk and K were significantly different between IAEC and EH, and the values of ADC, D, f, DDC, Dk and K were significantly different between IAEC and a thickened endometrium. For differentiation between IAEC and EH and between IAEC and a thickened endometrium, K was the most effective parameter, with an AUC of 0.890 and 0.841, respectively. The values of ADC, D, DDC and K were different between EH and a thickened endometrium, but the differential efficacy was poor, with all AUCs being < 0.70 (Table 4).

**Table 4:** Comparison of subgroups

parameters	Stage IA EC vs. EH			Stage IA EC vs.TE			EH vs. TE		
	t/Z	*p*	AUC	t/Z	*p*	AUC	t/Z	*p*	AUC
ADC (× 10^−3^mm^2^/s)	3.23	0.001^a^	0.675	5.51	< 0.001^a^	0.537	2.50	0.012 ^a^	0.653
D (× 10^−3^mm^2^/s)	6.33	< 0.001^a^	0.843	3.63	< 0.001^a^	0.783	2.71	0.007 ^a^	0.665
D* (× 10^−3^mm^2^/s)	1.49	0.136 ^a^	–	1.53	0.127 ^a^	–	1.62	0.106 ^a^	–
f (%)	0.87	0.384 ^a^	–	4.05	< 0.001^a^	0.578	0.20	0.842 ^a^	–
DDC (× 10^−3^mm^2^/s)	5.23	< 0.001^a^	0.783	0.61	< 0.001^a^	0.708	2.41	0.016 ^a^	0.647
α	1.08	0.278 ^a^	–	2.75	0.006 ^a^	0.532	0.48	0.633 ^a^	–
Dk (× 10^−3^mm^2^/s)	3.97	< 0.001^a^	0.715	5.51	< 0.001^a^	0.641	1.90	0.057 ^a^	–
K	9.16	< 0.001^b^	0.890	7.943	< 0.001^b^	0.841	2.40	0.019^b^	0.658

*ADC* apparent diffusion coefficient, *D* true diffusion coefficient, D* pseudodiffusion coefficient, *f* the perfusion fraction, *α* diffusion heterogeneity index, *DDC* distributed diffusion coefficient, *Dk* diffusion coefficient, *K* diffusion kurtosis, *a* Z, *b* t, *EC* endometrial carcinoma, *EH* endometrial hyperplasia, *TE* thickened endometrium

## Discussion

Magnetic resonance imaging plays an important role in the diagnosis and differential diagnosis of endometrial disorders. For advanced diseases, MRI is sensitive in detecting the interruption and extent of the myometrium. However, for early-stage diseases, especially those without obvious evidence of myometrium invasion on MRI, the imaging features of benign and malignant lesions often overlap. With advancements in MR sequences, some recent studies have focused on diffusion MRI for the evaluation of uterine cavity lesions (Takeuchi et al. 2005). In these studies, the diffusion images were found to be different when comparing endometrial cancer and normal endometrium or benign lesions; however, most of these studies focused on late-stage endometrial cancers with obvious invasion of the myometrium, which are easy to detect on MRI. In this study, we focused on the differential diagnosis of IAEC from BELs, which represent the most challenging detections for both radiologists and gynaecologists.

The values of ADC, D, DDC and Dk can all be used to reflect the restricted diffusion of water molecules in tissue and are mainly influenced by cell density. The apparent diffusion coefficient is the default measure of the mean diffusivity of water in tissue derived from the mono-exponential DWI model. While it is easy to obtain, ADC does not indicate the true diffusion value because it is also affected by the perfusion. The apparent diffusion coefficient was reported in the literature to have some ability to distinguish EC from BELs, but its efficacy for diagnosing early-stage EC is limited. In our study, the efficacy of ADC was low, with an AUC of 0.601, suggesting that advanced diffusion models are needed. Our study reveals that, compared with the other diffusion parameters, D had the highest diagnostic efficiency. This may be related to the fact that the value of D excludes the effect of microcirculation (Iima and Bihan 2016). Both DDC and Dk are regarded as composites of individual ADCs with different distributions and directions, respectively; theoretically, both can reflect the diffusion of water molecules more accurately than the ADC (Yamada et al. 2019). However, in our study, the diagnostic efficiency of DDC or Dk was not higher than ADC, which was inconsistent with previous studies (Meng et al. 2021a), possibly due to the difference in study groups. Intravoxel incoherent motion is a functional MRI method that can simultaneously reflect the capillary movement of water molecules (diffusion) and blood circulation (perfusion) in tissue. The D and D* values derived from the bi-exponential IVIM model reflect the diffusion of water molecules and micro-perfusion separately. The IVIM method was shown to be useful in the assessment of the biological behaviour of EC (Meng et al. 2021a), as well as the correlation of cell proliferation with antigen Ki-67 (Li et al. 2021) and the microsatellite instability status of EC (Ma et al. 2023; Bhosale et al. 2017). In our study, the D value of the IAEC group was lower than that of the BEL group, whereas the D* value of the IAEC group was higher compared with the BEL group. The difference in D* between the groups indicated that microperfusion was not negligible and should be further studied. The stretched exponential model has been developed to simultaneously quantify diffusion and tissue heterogeneity. The DDC parameter can reflect the diffusion movement status of water molecules in tissue. Zhang et al. found that DDC could be used to predict the grading of EC, and DDC outperformed ADC (Zhang et al. 2020). The α parameter provides information about the intravoxel water diffusion heterogeneity. A lower α value indicates a higher intravoxel diffusion heterogeneity caused by multi-exponential signal attenuation. Several previous studies have demonstrated that diffusion index α correlates with histological heterogeneity (Zhang et al. 2023; Liu et al. 2015). However, in our study, α did not show a significant difference between malignant and benign groups, presumably as our study focused on IAEC.

Diffusion kurtosis imaging is a method to quantify the deviations from free-water diffusion by estimating diffusion kurtosis from DWI images acquired at high b-values, compared with standard DWI protocols. A K-value derived from the DKI model is an extra parameter that can be used to describe the water molecular diffusivity beyond ADC and Dk. Diffusion kurtosis is a parameter without units, signifying the excess kurtosis compared with a mono-exponential Gaussian model; a larger value of K indicates a greater deviation of diffusion from perfectly Gaussian behaviour (Jensen et al. 2005; Jensen and Helpern 2010). An elevation in K was considered to represent cancer with reduced diffusion and increased complexity. Chen et al. found that DKI can be used to distinguish high-grade from low-grade ECs, and K was the best parameter in this regard, with an AUC of 0.891 (Chen et al. 2017). Yue et al. found that the value of K was higher in the EC than in the normal endometrium group; the AUC of the K value was 0.93, higher than that of Dk and ADC (Yue et al. 2019). The Dk value was obtained using the diffusion value from non-Gaussian distribution corrections, which reflect the overall diffusion and diffusion resistance of water molecules (Granata et al. 2020). The diffusion coefficient has been used to evaluate early-stage EC in several studies. Meng et al. (Meng et al. 2021a, 2021b) showed that Dk could be used to reflect risk stratification and histological features. In some research, Dk showed higher efficacy than ADC, which may be related to the fact that Dk was calculated by considering the restricted diffusion of water molecules in all directions and, therefore, could assess the diffusion of water molecules more accurately than ADC (Jin et al. 2022; Yue et al. 2019). Tian et al. found that both Dk and K were useful in the differential diagnosis of IAEC from endometrial polyps (Tian et al. 2023). The results from our study are consistent with those of previous studies. We found that the K-value in patients with IAEC was higher and Dk was lower than those in patients with BELs, which could be explained by the fact that EC tumour cells proliferate more actively and are more complex than benign lesion cells. Our result is consistent with the previous study; the AUCs of K and Dk were 0.864 and 0.675, respectively, slightly lower than those in the literature. However, the participants in our study were different from those in previous studies, as we focused on the differentiation of IAEC from BELs, which can be difficult for radiologists to establish. Our research also suggests that K performed better than Dk in differential diagnoses, which is consistent with previous studies.

This study had several limitations. First, some of the pathological diagnoses were established by endometrial curettage and biopsy, which may be subject to sampling error. However, such situations are unavoidable, given that a hysterectomy is not warranted for most benign pathologies. Second, all diffusion models used in this study were based on the same series of multiple b-values. Although there were suggestions for the tailored selection of different b-values for diverse advanced DWI models, no consensus was reached. Thus, we decided to use the same series to allow for easy comparison. However, this conclusion should be confirmed in a larger cohort of patients in future prospective studies.

In conclusion, this study compared the diagnostic efficacy of different diffusion models in differentiating IAEC from BELs. Among all diffusion parameters, K showed the best performance and was the only independent predictor; thus, DKI could be used as a non-invasive diagnosis method in clinical practice.

## Supplementary Information

Below is the link to the electronic supplementary material.

Supplementary file1 (DOCX 16 KB)

## Data Availability

All data generated or analysed during this study are included in this article. Further enquiries can be directed to the corresponding author.

## Abbreviations

EC: Endometrial carcinoma
EH: Endometrial hyperplasia
BEL: Benign endometrial lesion
DWI: Diffusion-weighted imaging
ADC: Apparent diffusion coefficient
IVIM: Intravoxel incoherent motion
DKI: Diffusion kurtosis imaging
EPI: Echo planar imaging
D: True diffusion coefficient
D*: Pseudodiffusion coefficient
f: The perfusion fraction
α: Diffusion heterogeneity index
DDC: Distributed diffusion coefficient
Dk: Diffusion coefficient
K: Diffusion kurtosis
VOI: Volumn of interest
ICC: Intra-class correlation coefficient
ROC: Receiver operating characteristic
AUC: Area under the ROC curve
