# Supplementary material for: A comparative study of mono-exponential and advanced diffusion-weighted imaging in differentiating stage IA endometrial carcinoma from benign endometrial lesions
Source: J Cancer Res Clin Oncol. 2024 Mar 20;150(3):141. doi: 10.1007/s00432-024-05668-8 (PMC10951008; doi:10.1007/s00432-024-05668-8)
Supplement: Supplementary file 1 — Supplementary file1 (DOCX 16 KB) [file 432_2024_5668_MOESM1_ESM.docx]

Table S1 Inter-observer consistency of parameters

| Diffusion model | parameter | Reader 1 | Reader 2 | ICC |
| --- | --- | --- | --- | --- |
| Mono-exponential | ADC(×10-3mm2/s) | 1.08 (0.97, 1.20) | 1.08 (0.96, 1.21) | 0.997 |
| Bi-exponential | D(×10-3mm2/s) | 0.78 (0.68, 0.87) 4.67 | 0.77 (0.65, 0.88) | 0.973 |
|  | D^*^(×10-3mm2/s) | (4.03, 4.87) | 4.52 (3.94, 4.98) | 0.912 |
|  | f (%) | 2.48 (2.20, 2.83) | 2.46 (2.17, 2.79) | 0.967 |
| stretched-exponential | DDC (×10-3mm2/s) | 1.17 (1.03, 1.31) | 1.16 (1.02, 1.32) | 0.989 |
|  | α | 0.63 (0.58, 0.67) | 0.52 (0.46, 0.65) | 0.761 |
| DKI | Dk (×10-3mm2/s) | 1.50 (1.36, 1.64) | 1.49 (1.36, 1.63) | 0.989 |
|  | K | 0.87±0.18 | 0.87±0.19 | 0.984 |

Note: ADC: apparent diffusion coefficient; D: true diffusion coefficient; D^*^: pseudodiffusion coefficient; f: the perfusion fraction; α: diffusion heterogeneity index; DDC: distributed diffusion coefficient; Dk: diffusion coefficient; K: diffusion kurtosis; DKI: diffusion kurtosis imaging.

Table S2 Comparison of AUCs

| parameter | ADC | D | D^*^ | DDC | Dk | K |
| --- | --- | --- | --- | --- | --- | --- |
| ADC | — | ＜0.001^*^ | 0.555 | ＜0.001^*^ | ＜0.001^*^ | ＜0.001^*^ |
| D | ＜0.001^*^ | — | 0.001^*^ | ＜0.001^*^ | ＜0.001^*^ | 0.014^*^ |
| D^*^ | 0.555 | 0.001^*^ |  | 0.076 | 0.55 | ＜0.001^*^ |
| DDC | ＜0.001^*^ | ＜0.001^*^ | 0.076 | — | ＜0.001^*^ | ＜0.001^*^ |
| Dk | ＜0.001^*^ | ＜0.001^*^ | 0.55 | ＜0.001^*^ | — | ＜0.001^*^ |
| K | ＜0.001^*^ | 0.014^*^ | ＜0.001^*^ | ＜0.001^*^ | ＜0.001^*^ |  |

Note: ADC: apparent diffusion coefficient; D: true diffusion coefficient; D^*^: pseudodiffusion coefficient; DDC: distributed diffusion coefficient; Dk: diffusion coefficient; K: diffusion kurtosis; AUC: area under the ROC curve; ^*^: *p*＜0.05
